# Long-Term Impact of Community Psychiatric Care on Quality of Life amongst People Living with Schizophrenia: A Systematic Review

**DOI:** 10.3390/healthcare12171750

**Published:** 2024-09-02

**Authors:** Jožica Černe Kolarič, Anja Plemenitaš Ilješ, Darja Kraner, Vida Gönc, Mateja Lorber, Nataša Mlinar Reljić, Zvonka Fekonja, Sergej Kmetec

**Affiliations:** 1 Faculty of Health Sciences, University of Maribor, Žitna ulica 15, 2000 Maribor, Slovenia; vida.goenc@um.si (V.G.); mateja.lorber@um.si (M.L.); natasa.mlinar@um.si (N.M.R.); zvonka.fekonja@um.si (Z.F.); sergej.kmetec1@um.si (S.K.); 2 Department of Psychiatry, University Clinical Centre Maribor, Ljubljanska ulica 5, 2000 Maribor, Slovenia; anja.plemenitas@ukc-mb.si; 3 Centre for Adult Mental Health, Community Healthcare Center, Ulica talcev 9, 2000 Maribor, Slovenia; darja.kraner@zd-mb.si

**Keywords:** schizophrenia, quality of life, community, psychiatric care, patient, systematic review

## Abstract

The review examines the long-term impact of community psychiatric care on improving the quality of life of people with schizophrenia. It addresses the global burden of this disorder and the need for effective community-based care strategies. A systematic literature search was conducted in databases such as CINAHL, Medline, Web of Science, Sage and ScienceDirect, with the search lasting until March 2024 and following the PRISMA guidelines. The inclusion criteria targeted studies that addressed the long-term effects of community mental health care for people aged 18 years and older with schizophrenia and included both quantitative and qualitative research designs. Studies unrelated to the research question or with significant methodological flaws were excluded. The risk of bias was assessed using GRADE and GRADECERqual, in addition to critical appraisal using the Joanna Briggs Institute (JBI) checklists. Independent screening and data extraction was performed, with results summarised by thematic analysis. In total, 11 studies met the inclusion criteria and included cross-sectional, cohort, qualitative and randomised controlled trial designs. The results showed that community psychiatric care significantly improves the quality of life, well-being and social integration of people with schizophrenia. Effective interventions identified include psychoeducation, cognitive behavioural therapy, social skills training and individualised care plans. However, challenges such as limited resources, labour shortages and social stigma, particularly in low-income neighbourhoods, were also identified. This study highlights the importance of continuous, personalised, multidisciplinary community-based care for sustainable mental health outcomes. Further research is recommended to investigate the long-term impact and strategies to overcome implementation challenges.

## 1. Introduction

Schizophrenia, affecting approximately 20 million individuals globally, is a debilitating mental disorder characterised by profound disruptions in thinking, behaviour, emotions and cognition [1]. The illness is a heavy burden for people living with schizophrenia. They are often confronted with social stigmatisation, which leads to social isolation, difficulties in maintaining relationships and a poorer quality of life (QoL) [2]. Consequently, improving the QoL for people with schizophrenia is a critical treatment goal [3,4].

People living with schizophrenia face major challenges, e.g., in finding and maintaining employment, socialising and leading an independent life due to positive symptoms (e.g., delusions and hallucinations) and negative symptoms (e.g., emotional blunting and lack of motivation) [5]. Cognitive impairments such as memory lapses and attention problems, as well as restlessness and depressive symptoms, further exacerbate these challenges [6]. The frequent recurrence of symptoms often leads to repeated hospitalisation and psychiatric emergency admissions, as in 28–30% of cases [7]. The World Health Organisation’s (WHO) 2013 Action Plan aims to integrate mental health into community-based care, focusing on accessibility, continuity and integrating community psychiatric care into primary care [5]. Community psychiatric care, implemented in various countries, aims to address the needs of individuals with severe mental disorders within their environments. This multidisciplinary approach involves psychiatrists, social workers, psychologists, nurses and occupational therapists [6,8], providing comprehensive and continuous care [9].

Community psychiatric care has proven to be very beneficial, especially in the long term, including better support and understanding for people with schizophrenia and their families. Healthcare professionals in community psychiatric care play a central role in psychoeducation and relapse prevention, contributing to better health outcomes [10]. It also includes the monitoring and treatment of people living with schizophrenia in their own homes and addresses their needs on a long-term basis [11]. This approach includes proactively supporting vulnerable people, promoting meaningful integration into family and society, and improving adherence to treatment and social skills [1,12].

Community psychiatric care faces challenges despite these benefits, particularly in low-income countries with limited resources and labour shortages [5]. Nevertheless, comprehensive interventions, including psychoeducation, cognitive behavioural therapy and anti-stigma strategies, can reduce discrimination and improve clinical outcomes [13]. The combination of psychosocial and pharmacological support leads to the best results in functional recovery and symptom management [14]. Those who receive community psychiatric care are 20–50% less likely to discontinue treatment, while those who do not receive such care are 40–80% more likely to discontinue treatment [15]. However, the long-term effectiveness of community psychiatric care in different socioeconomic settings has yet to be sufficiently researched. Most studies have focused on short-term outcomes, leaving a gap in an understanding of the sustainable benefits and challenges that emerge over time. This review aimed to address these gaps and provide insights into the long-term impact of community psychiatric care on improving the QoL of people with schizophrenia.

## 2. Materials and Methods

A systematic review was conducted and registered with PROSPERO (ref. number: 567236), following Page’s guidelines [16] for identifying, screening, assessing eligibility and including students.

### 2.1. Research Question

The research question was developed using the Population, Intervention, Outcome (PICO) approach described by Melnyk and Fineout–Overholt [17] and is was “How effective (O) is community psychiatric care (I) compared with standard inpatient psychiatric care (C) in terms of improving long-term outcomes (reducing relapse rates, improving treatment adherence and improving the overall quality of life) in people with schizophrenia (P)?”

### 2.2. Search Strategy

A comprehensive search strategy was developed using English search terms and Boolean operators (AND/OR) to capture relevant literature on community psychiatry and schizophrenia. The final search terms were (meaning* OR importance* OR impact*) AND (“community psychiatry*” OR “community psychiatric”) AND (treatment OR care OR service) AND (patient OR (people OR individual)) AND carer AND schizophrenia. The CINAHL, Medline, Web of Science, Sage and ScienceDirect databases, which contain extensive medical and health-related research indexes, were searched to ensure thorough coverage. Additionally, a hand search for grey literature was conducted. The search was limited to research published up to March 2024 in English, German and Slovenian. Consistent search terms, restrictions, and inclusion and exclusion criteria were applied across all databases. Supplementary Table S1 contains the detailed search results for each database.

### 2.3. Eligibility Criteria

Eligibility criteria were defined to ensure the relevance and quality of the review. Studies were included that examined the long-term effects of community psychiatric care for people with schizophrenia aged 18 years and older, using both quantitative and qualitative research designs. Studies based on quantitative, qualitative and mixed methods were considered. Exclusion criteria included studies that were not directly related to our research question or those with significant methodological flaws. Studies involving people under the age of 18, people who had not been diagnosed with schizophrenia or the general population were excluded. In addition, studies that did not focus on community psychiatric care or its long-term effects on people with schizophrenia were not included. Systematic reviews, other review articles, conference papers and editorials were also excluded from our review.

### 2.4. Critical Assessment

The selected studies were categorised into eight levels of evidence based on the framework [18], with systematic reviews or meta-analyses of randomised trials representing the highest level. To ensure the reliability of the evidence in this systematic review, the quality of each study was assessed using the GRADE approach [19] for quantitative studies and the GRADECERQual approach for qualitative studies. The GRADE approach assesses the quality of evidence based on areas such as risk of bias, inconsistency, indirectness, imprecision and publication bias. The GRADECERQual approach [20] assesses confidence in qualitative evidence based on methodological limitations, coherence, the data’s adequacy and relevance. The included studies were assessed using the Joanna Briggs Institute (JBI) checklists specific to their design: the cross-sectional analytical study checklist [21] with eight questions for cross-sectional studies, the cohort study checklist [21] with 12 questions for cohort studies, the randomised controlled trials (RCT) checklist [22] with 12 questions for RCTs, the qualitative research checklist [23] with 10 questions for qualitative studies and the quasi-experimental study checklist [24] with 9 questions for non-randomised or quasi-experimental studies. Each “yes” answer on the checklist received one point, while “no” and “unclear” received zero points. After scoring, the total score and percentage were calculated for each trial. According to the recommendation of Camp and Legge [25], the studies were categorised into four quality groups: low quality (60–69%), moderate quality (70–79%), high quality (80–89%) and excellent quality (90 or higher). To be included in the review process, each study had to achieve a compliance rate of at least 70%.

### 2.5. Data Extraction and Synthesis of the Data

The first author initially performed data extraction using predefined criteria (such as authors, year, country, purpose, etc.), followed by a review from the other authors. Disagreements were resolved through discussion and consensus to ensure accuracy and reliability.

Data synthesis was based on the content framework of Elo and Kyngäs [26]. The first author read through the text line by line and identified free codes for each study included. These free codes were then categorised into created subcategories, and analysed and compared using the MAXQDA Analytics Pro program to develop a secondary subtheme and a main theme. The other authors then reviewed the thematic synthesis, and any disagreements were resolved through discussion and consensus.

### 2.6. Outcome Measures and Variables

The review sought data on several primary and secondary outcomes to comprehensively assess the long-term impact of community mental health services on people with schizophrenia. The primary outcomes included QoL and well-being, measured using validated scales such as WHOQOL-BREF (World Health Organisation Quality of Life), Clinical Global Impression-Schizophrenia (CGI-SCH), and the Positive and Negative Syndrome Scale (PANSS). Secondary outcomes included social integration, severity of symptoms, adherence to treatment and functioning, assessed using Global Assessment of Functioning (GAF) scales, the Brief Psychiatric Rating Scale (BPRS) and records of adherence to medication.

Additional variables included demographic information (age, gender, socioeconomic status, educational level), study characteristics (design, sample size, duration of follow-up, geographic location) and details of the intervention (type, frequency, duration and therapeutic components such as psychoeducation and cognitive behavioural therapy). The results were summarised using effect measures such as the mean difference (MD) and standardised difference (SD) for pooled data, and risk ratios (RR) for binary outcomes.

To determine which outcomes to collect, each study was searched for all outcomes that matched the outcome domain, including all reported measures, time points and analyses. If multiple measures or time points were available for the same outcome, the most comprehensive and relevant data were selected on the basis of predefined criteria, prioritising the longest follow-up time and the most rigorous methodological quality. Data extraction was performed independently by two reviewers to ensure consistency and accuracy. Any discrepancies were clarified by discussion or consultation with a third reviewer.

## 3. Results

### 3.1. Selection of Relevant Paper

**Figure 1:**
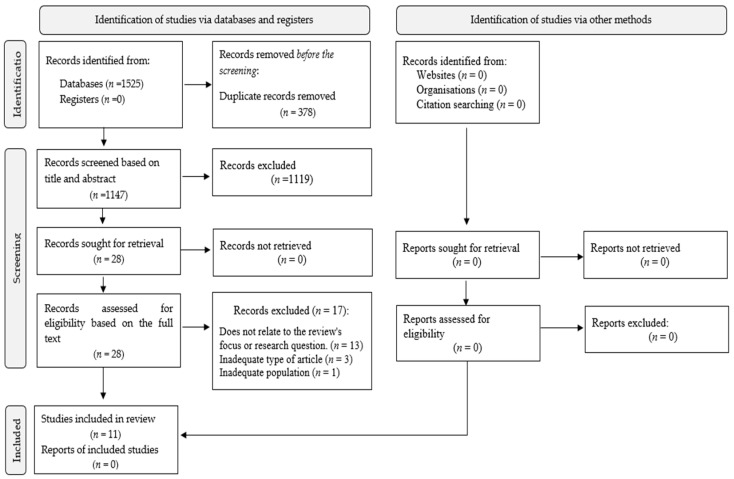
PRISMA flow diagram of selecting the studies.

The search strategy resulted in 1525 records across the databases: CINAHL (*n* = 3), Medline (*n* = 9), Sage (*n* = 756), ScienceDirect (*n* = 753) and Web of Science (*n* = 4) (see Supplementary Table S1) for search results in each database). A hand search for grey literature was also conducted, but no records were found. After removing 378 duplicates, 1147 records remained for further review. After screening of the titles and abstracts, 1119 records were excluded because they did not fulfil the inclusion criteria. The full texts of the remaining 28 studies were screened, resulting in the exclusion of 17 studies. Ultimately, 11 studies were included in the final analysis and synthesis. All included studies scored at least 70% in the quality assessment, which ensured their reliability for inclusion in the review. A detailed list of the excluded studies and the reasons for exclusion can be found in Supplementary Table S2. Disagreements during the selection process were resolved by discussion, with a third author consulted if a consensus could not be reached. The selection process is illustrated in Figure 1.

### 3.2. Characteristics of the Included Studies

The 11 studies had a variety of research designs, including five cross-sectional studies, two cohort studies, two qualitative studies and two randomised controlled trials. The geographical distribution of the studies included Taiwan, Nigeria, Switzerland, Poland, Thailand, Turkey, China, Greece and Germany. The sample sizes ranged from 28 to 471 participants. These studies focused on individuals with schizophrenia treated in various settings, such as hospitals, community psychiatric centres and rehabilitation centres, with an emphasis on community psychiatric care. Table 1 shows the detailed characteristics of each study included.

### 3.3. Critical Assessment and Level of Evidence of Included Papers

The quality of the included studies ranged from moderate to excellent. Most studies were highly compliant with methodological standards. However, some limitations were noted, particularly regarding the blinding of participants in RCTs and including confounding factors in cross-sectional studies. According to Polit and Beck’s [18] criteria for the level of evidence, the studies were categorised as follows: Li et al. [12] and Luo et al. [32] were at Level 2 (RCT), Schöttle et al. [33] and Golay et al. [6] were at Level 3 (cohort studies), and Elegbede et al. [4], Chen et al. [27], Peritogiannis and Nikolaou [30], Kurt and Erşan [29] and Hat et al. [28] were at Level 5 (cross-sectional studies). Juntapim and Nuntaboot [31] and Zheng et al. [1] represented Level 7 (qualitative studies). According to the GRADE assessment, three studies [12,32,33] were categorised as high and six [4,6,27,28,29,30] as moderate. With regard to the GRADE-CERQual assessment, two studies [1,31] were categorised as high. A summary of the critical appraisals and the GRADE/GRADE-CERQual assessments can be found in Table 2 (Supplementary Table S3: GRADE/GRADE-CERQual rating of the included studies).

### 3.4. Primary Outcomes

A comprehensive analysis was conducted to determine the overall impact of community psychiatric care on individuals with schizophrenia. Table 3 summarises the quantitative results, including key aspects such as the interventions, instruments used, number of participants and statistical measures.

#### 3.4.1. Quality of Life (QoL)

All included studies measured QoL as a primary outcome, using various validated scales such as WHOQOL-BREF and PANSS. For example, Li et al. [12] reported that participants in the community-based intervention group had a mean QoL score of 60 (SD = 10), with a significant reduction in symptoms (RR = 0.75, *p* < 0.001). Similarly, Elegbede et al. [4] found that community psychiatric care significantly improved QoL compared with inpatient care (RR = 1.05, *p* = 0.020, M = 75, SD = 15). These findings consistently demonstrated the positive impact of community psychiatric care on QoL among individuals with schizophrenia.

#### 3.4.2. Relapse Rates

Two randomized controlled trials [12,32] specifically evaluated relapse rates as a primary outcome. The community-based intervention groups in these studies exhibited significantly lower relapse rates compared with standard inpatient treatment. Li et al. [12] reported a relapse rate of 20% in the intervention group compared with 50% in the control group (RR = 0.80, *p* < 0.001), underscoring the effectiveness of community psychiatric care in reducing relapses.

### 3.5. Secondary Outcomes

#### 3.5.1. Adherence to Treatment

Adherence to treatment was another key outcome reported in several studies. Schöttle et al. [33] found that rates of adherence to treatment increased from 25.2% to 78.3% (*p* = 0.005) in participants receiving community psychiatric care. This improvement was attributed to the personalized and continuous support provided in community settings, which facilitated better adherence to treatment regimens.

#### 3.5.2. Social Functioning

Improvements in social functioning were consistently reported across the studies. Kurt and Erşan [29] compared social functioning in patients receiving care at community mental health centres (CMHCs) with those treated in hospital settings. The CMHC group had significantly higher social functioning scores (M = 65, SD = 13, *p* = 0.015) and lower rates of hospitalization (RR = 0.85, *p* < 0.001). These findings highlight the role of community-based interventions in enhancing social integration and functional outcomes.

### 3.6. Additional Outcomes

#### 3.6.1. Patients’ Satisfaction

Several studies assessed patients’ satisfaction as an additional outcome. For instance, Chen et al. [27] used the Picker Institute’s PCC domains to measure satisfaction among patients with schizophrenia. The study found that emotional support and effective communication were key predictors of higher satisfaction, with a mean satisfaction score of 68 (SD = 14, *p* = 0.008) in the community care group.

#### 3.6.2. Symptom Severity

Reductions in symptoms’ severity was another important outcome evaluated. Luo et al. [32] reported significant reductions in symptom severity among participants in the Assertive Community Treatment (ACT) program, as measured by GAF and CGI-SCH scores (OR = 1.20, *p* = 0.002, M = 70, SD = 12). These results underscored the effectiveness of community-based interventions in managing the clinical symptoms of schizophrenia.

### 3.7. Synthesis of Qualitative Data

**Figure 2:**
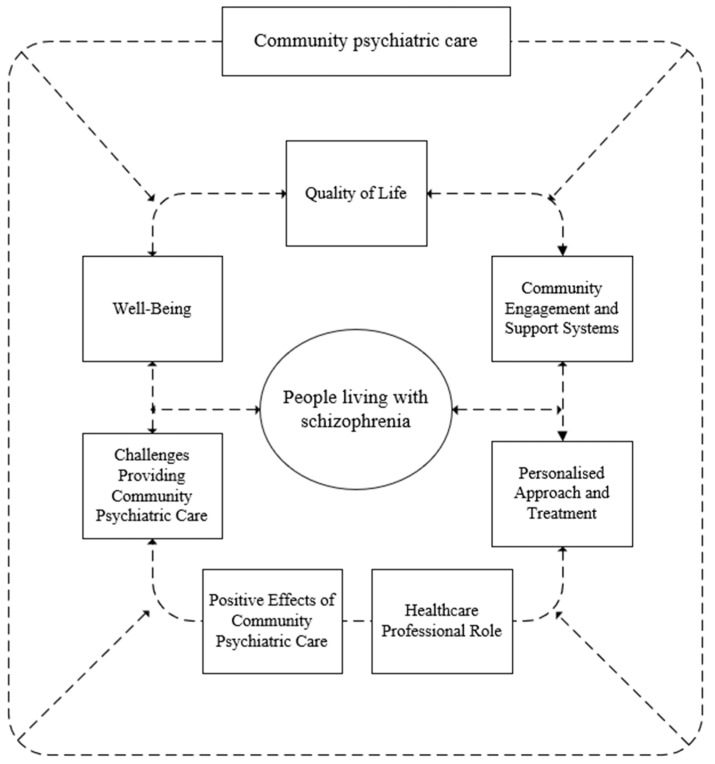
Display of a synthesis of the studies included.

The synthesis also included a qualitative analysis (including the qualitative and quantitative aspects of the studies included), in which the articles (*n* = 11) were coded line by line, resulting in the formation of free codes (*n* = 209). These were further categorised into subcategories (*n* = 7) and finally combined into one main category: “the impact of community mental health services on people with schizophrenia” This is visually represented in Figure 2, which shows the seven main subcategories contributing to the main category.

The main category, “long-term impact of community psychiatric care on people living with schizophrenia,” included the following subcategories: (1) QoL, (2) well-being, (3) community engagement and support systems, (4) role of healthcare professionals, (5) individualised approach and treatment, (6) positive impact of community mental health care, and (7) challenges in providing community mental health care.

#### 3.7.1. Quality of Life

QoL is a multifaceted concept encompassing various domains such as physical health, psychological well-being, social relationships and environmental conditions. The included studies [1,4,6,12,27,33] consistently highlighted the important long-term impact of community psychiatric care in improving the QoL and functioning of people with severe mental illnesses such as schizophrenia. Chen et al. [27] emphasised the importance of PCC, including emotional support and effective communication. People living with schizophrenia who felt understood and supported by their healthcare providers reported higher levels of satisfaction and an overall better QoL. Elegbede et al. [4] compared the QoL of people living with schizophrenia in community psychiatric care with the QoL of people in a psychiatric hospital. The results showed that people living with schizophrenia participating in a community psychiatric centre had significantly higher QoL scores. Community psychiatric care provides a supportive and less restrictive environment, contributing to better social integration, greater autonomy and better mental health outcomes. Golay et al. [6] found that people living with QoL receiving community psychiatric care improved sustainably over time. Consistent and individualised support in the community helped people living with schizophrenia manage their symptoms better and engage in meaningful activities, improving their QoL.

#### 3.7.2. Well-Being

Well-being encompasses various aspects of mental health, including emotional stability, self-esteem, social functioning and effective coping with daily life. Research has consistently shown that community psychiatric care significantly improves the well-being of people with severe mental illnesses such as schizophrenia. This improvement is attributed to key factors such as comprehensive intervention programmes, structured support systems and active involvement in the community. Li et al. [12] found that comprehensive intervention programmes have a significant long-term impact on well-being. These programmes, which include psychoeducation, cognitive behavioural therapy (CBT) and social skills training, have been shown to increase self-esteem and reduce internalised stigma in people living with schizophrenia. Psychoeducation gives people living with schizophrenia a better understanding of their illness and provides them with knowledge and coping strategies. CBT helps to overcome negative thoughts and behavioural patterns and develop a more positive self-image. Social skills training enables people living with schizophrenia to interact more effectively in their environment, reduce feelings of isolation and strengthen social support networks. Consistent and personalised support in the community plays a crucial role in maintaining these positive outcomes and promoting ongoing well-being.

#### 3.7.3. Community Engagement and Support Systems

Community engagement and support systems are critical to the mental health and well-being of people with serious mental illness. These elements promote social integration, provide emotional and practical support, and improve the effectiveness of psychiatric care. Structured, supportive community psychiatric care, including regular follow-up, tailored care plans and access to resources, significantly enhances the QoL of people living with schizophrenia, reduces hospital admissions and provides stability and security [28].

Juntapim and Nuntaboot [31] emphasised the benefits of community involvement and psychoeducation. Active participation in community activities and educational programmes improves self-management skills and emotional health. These activities provide meaningful interactions and opportunities to build social networks, which are essential for emotional support and resilience. Psychoeducation offers people living with schizophrenia with the knowledge and skills to manage their illness independently, promoting self-determination and self-efficacy. Feeling valued and included in society increases well-being [29,31]. People living with schizophrenia who attend community mental health centres (CMHCs) show significant improvements in social skills, community participation and overall social functioning. CMHCs offer social skills training, vocational rehabilitation and recreational activities that contribute to developing life skills and promoting social integration. The continuum of care and crisis intervention services provided by CMHCs help to reduce hospitalisations and maintain community stability [29]. Active participation in community activities and support systems improves social skills, provides meaningful involvement and promotes a sense of belonging, all contributing to a better QoL [4]. Strong community support systems, including peer support groups and family involvement, are critical to the sustainability of rehabilitation outcomes. Peer support groups provide a safe space for sharing experiences and developing coping strategies. In contrast, family involvement offers emotional support and reinforces positive behaviours that are essential for sustainable rehabilitation and improved mental health outcomes [1].

By offering comprehensive services and intensive support and fostering strong community relationships, these systems significantly improve social functioning, reduce hospitalisation rates and promote long-term recovery and stability for people with severe mental illness [29,31].

#### 3.7.4. Personalised Approach and Treatment

Community psychiatric care involves multi-faceted treatment approaches that combine therapeutic interventions, personalised care plans and community-based support systems to treat severe mental illnesses such as schizophrenia effectively. Personalised interventions address individual needs and preferences and improve QoL [27]. Hat et al. [28] found that community psychiatric care improves symptom management and overall well-being through holistic, ongoing support, regular monitoring and access to resources, reduces hospitalisation rates and promotes long-term stability.

Elegbede et al. [4] observed better outcomes for people living with schizophrenia in community psychiatric care compared with those in hospitals due to personalised care and frequent interactions. Customised care plans lead to more effective disease management. Kurt and Erşan [29] also reported better social functioning and fewer hospitalisations with comprehensive, continuous care in community psychiatric care. The multidisciplinary approach involving psychiatrists, psychologists, social workers and other health professionals is crucial for treating complex people living with schizophrenia.

Li et al. [12] highlighted the effectiveness of multifaceted intervention programmes, including psychoeducation, cognitive behavioural therapy (CBT) and social skills training. Psychoeducation helps people living with schizophrenia and their families understand the illness, treatment options and coping strategies, thereby reducing stigmatisation and promoting adherence to treatment. CBT addresses negative thinking and behaviour patterns, improves mental health and reduces symptoms. Social skills training improves the interactions in the community of people living with schizophrenia, which enhances social functioning and QoL. Integrating these interventions into the community provides comprehensive support that is critical to managing mental illness. Luo et al. [32] emphasised that ACT reduces rates of relapse and readmission. ACT’s strong support network and continuous monitoring enable early detection and intervention, significantly reducing rates of relapse and hospitalisation compared with standard treatment. The ACT teams’ hands-on approach and comprehensive support are key to maintaining stability and promoting recovery.

Juntapim and Nuntaboot [31] found that participation in community activities and educational programmes improved self-management skills and emotional well-being. These programmes provide a supportive environment for building social networks, developing coping strategies and a better understanding of conditions, highlighting the importance of social support and community engagement for recovery and treatment outcomes.

#### 3.7.5. Healthcare Professionals’ Role

Health professionals play a crucial role in the success of community psychiatric care by providing personalised, continuous and compassionate support to people with severe mental illness. The studies reviewed highlighted how health professionals contribute to better outcomes for people living with schizophrenia through effective communication, emotional support and tailored interventions. Chen et al. [27] stressed the importance of effective communication and emotional support from healthcare professionals and found that people living with schizophrenia who felt understood and supported reported higher levels of satisfaction and a better QoL. Healthcare professionals often act as counsellors, advocates and caregivers, building trust and relationships, which are essential for treatment adherence and positive therapeutic relationships. Elegbede et al. [4] found that personalised care, in which professionals actively listen and respond to the needs of people living with schizophrenia, improved satisfaction and treatment outcomes. Golay et al. [6] emphasised the role of healthcare professionals in providing various services such as crisis intervention, case management and therapeutic support. Their involvement ensured comprehensive, continuous care, which led to improved QoL and reduced hospitalisation. Adapting care plans to the changing needs of people living with schizophrenia is critical to the success of community psychiatric care.

Hat et al. [28] emphasised the role of healthcare professionals in structured community-based support systems. Regular follow-up, personalised care plans and continuous monitoring by healthcare professionals improved emotional stability and reduced hospital admissions. Proactive approaches, emotional support and prompt responses to concerns are key to effective community psychiatric care. Juntapim and Nuntaboot [31] discussed the influence of health professionals on community engagement and psychoeducation programmes. These professionals design and implement programmes that improve the self-management skills and emotional health of people living with schizophrenia. Involvement in community activities and educational programmes helps people living with schizophrenia to strengthen their resilience, develop coping strategies and better understand their illness.

#### 3.7.6. Positive Effects of Community Psychiatric Care

Community psychiatric care offers many long-term benefits for people with severe mental illness, particularly schizophrenia. Research shows that functioning, social integration and overall mental stability improve significantly due to the comprehensive, individualised and ongoing support provided in community psychiatric care.

Peritogiannis and Nikolaou [30] found that many people living with schizophrenia achieve satisfactory functioning despite persistent symptoms, which is promoted by the supportive environment of rural communities and mobile services of community psychiatric care. This environment favours social integration and functional recovery. Juntapim and Nuntaboot [31] observed that by actively participating in community activities, people living with schizophrenia improved their self-management, social skills and emotional well-being, which promoted recovery and reduced stigmatisation.

Kurt and Erşan [29] reported that community psychiatric care significantly improves social reintegration and the stability of mental health. Services such as vocational training, recreational activities and therapeutic support help people living with schizophrenia to develop life skills and self-confidence, which reduces hospitalisation rates and promotes long-term recovery. Chen et al. [27] and Elegbede et al. [4] emphasised the positive long-term impact of community psychiatric care on the satisfaction and QoL of people living with schizophrenia. Chen et al. [27] highlighted the role of emotional support and effective communication by healthcare professionals. Elegbede et al. [4] found that community psychiatric care performed better than hospitals regarding QoL, highlighting the importance of personalised care. Golay et al. [6] found sustained improvements in QoL and symptom management in community psychiatric care, with continuous and personalised care reducing relapse rates and increasing stability. Li et al. [12] found that a comprehensive approach to community psychiatric care reduced discrimination and stigmatisation. At the same time, Zheng et al. [1] emphasised the need to enrich the daily lives of people living with schizophrenia with social and recreational activities that give them hope, happiness and a sense of belonging.

#### 3.7.7. Challenges to Providing Community Psychiatric Care

The provision of community psychiatric care is faced with various challenges that impact the effectiveness and accessibility of community psychiatric care. These challenges are diverse and specific to different contexts, reflecting variations in healthcare systems, societal attitudes and resource availability across regions.

The study by Schöttle et al. [33] identified one of the main challenges was ensuring continuity in the utilisation of services and addressing the diverse needs of people living with schizophrenia or with severe mental illness. The study also highlighted the difficulty of maintaining high-quality care without integrated, specialised services. Similarly, Hat et al. [28] emphasised challenges such as the requirement for a robust support system involving family members and GPs and specialised forms of community psychiatric care that are not uniformly accessible in all regions. Similarly, Peritogiannis and Nikolaou [30] found that the lack of mental health facilities forces many people living with schizophrenia to rely on mobile mental health facilities, which are often overburdened. The study suggested that while these units play an important role, they cannot provide comprehensive care to all people living with schizophrenia due to their limited resources.

Furthermore, Zheng et al. [1] emphasised that despite extensive public education efforts, there is still a significant lack of awareness and understanding of mental health problems among the general public. This contributes to high levels of stigma and discrimination, which increases the self-stigmatisation of people living with schizophrenia and discourages them from seeking help and integrating into society. The study found that people living with schizophrenia often prefer the safety of rehabilitation centres or their families to the prejudices of society.

Ensuring the long-term sustainability of community psychiatry services is another crucial challenge. Golay et al. [6] emphasised that these models need to be continuously supported and adapted to meet the changing needs of people living with schizophrenia. They highlighted the importance of flexibility and responsiveness in community mental health care.

## 4. Discussion

The systematic review highlighted several critical aspects of community psychiatric care and their long-term impact on people living with schizophrenia. The key themes identified include (1) QoL, (2) well-being, (3) community engagement and support systems, (4) the role of healthcare professionals, (5) individualised approaches and treatment, (6) the positive effects of community psychiatric care and (7) challenges in the provision of community psychiatric care.

First, the results showed that community psychiatric care significantly improves the QoL and well-being of people with schizophrenia through a person-centred approach, emotional support and effective communication. Such care promotes social integration, autonomy and psychological stability, which are essential for long-term recovery. Comprehensive intervention programmes, personalised care plans and the active involvement of healthcare professionals are crucial for the effective treatment of severe mental illness. Community psychiatric support programmes such as psychoeducation, cognitive behavioural therapy (CBT) and social skills training have been shown to improve mental health and reduce symptoms, facilitating long-term recovery. In addition, the supportive environment provided by community psychiatric care, including mobile services, promotes social integration and functional recovery.

However, the review also highlighted some challenges that impact the effectiveness and accessibility of community psychiatric services. These challenges include ensuring continuity of care, addressing the diverse needs of people with schizophrenia, the limited availability of specialised services, restricted access to care and the widespread stigma and discrimination associated with mental illness.

Several studies, including those by Elegbede et al. [4], Li et al. [12], Zheng et al. [1], Puntis, Minichino [34] and Schöttle et al. [33], indicated that community psychiatric care contributes to better satisfaction of people living with schizophrenia. For example, Zheng et al. [1] found that community psychiatric care activities enrich the lives of people living with schizophrenia and meet their needs for socialisation and entertainment. Other studies, such as those by Killackey et al. [35], emphasised that the main benefit of community psychiatric care is to help people with schizophrenia develop the skills needed to live independently in their community. Community psychiatric care has been shown to help reduce hospitalisation and provide support for people living with schizophrenia to cope with difficulties in daily life, work and education [35,36]. It also offers an alternative to long-term hospitalisation, contributing to fewer involuntary admissions and shorter hospital stays, ultimately reducing the cost of overall care [4,6,29,32,33]. THE studies by Zheng et al. [1], Schöttle et al. [33], and Kurt and Erşan [29] showed that community psychiatric care can improve adherence to medication and increase compliance with treatment. Community psychiatric rehabilitation significantly improves the social functioning and skills of people living with schizophrenia [1,12,29]. People living with schizophrenia who received community psychiatric treatment had a shorter average time to re-employment, as more people living with schizophrenia were employed after 24 months of treatment [32,33]. Community mental health care reduced the frequency of negative symptoms and led to an overall improvement in clinical symptoms [12,29,33].

The report emphasised the importance of focusing on the specific needs of the community in which psychiatric care is provided. Utilising existing resources and collaborating with different sectors within the community are essential for successfully implementing community psychiatric care. The development of policy at a national level that supports the planning and development of community psychiatric care while enabling the mobilisation of social capital is also crucial [6,31]. Furthermore, a study by Thornicroft et al. [37] suggested that mental health care should balance community and hospital-based psychiatric care, with a significant proportion of care provided in the patient’s home. National governments should set targets to achieve adequate coverage, promote care that is both accessible and acceptable to users, and work to reduce stigmatisation and discrimination against people with mental illness [6,31]. Thoegersen et al. [38] and Schöttle et al. [33] pointed out that a community psychiatry approach helps to strengthen the relationship between the patient and the healthcare professional. Li et al. [12] stated that trust should be built with the patient and communication should be encouraged. Ådnanes et al. [39] stated that the therapeutic relationship is important for people living with schizophrenia, as it improves their engagement and helps them achieve their goals. In addition, Schöttle et al. [33] stated that psychotherapeutic attitudes and the intensive involvement of family members or significant others in the treatment process improve therapeutic engagement.

Further research could help people living with schizophrenia achieve a better QoL and greater satisfaction and reduce stigmatisation and discrimination. In the future, it would be worthwhile investigating how to improve the early detection of people living with schizophrenia and take timely action. It would be interesting to examine how support from family, friends, carers and the community affects treatment outcomes. It would also be worthwhile to explore and investigate effective public awareness and education strategies to reduce the stigmatisation of people living with schizophrenia. Further research is essential to improve community psychiatric care and ensure holistic care for people living with schizophrenia.

### 4.1. Advantages and Limitations of the Literature Review

Our review of community psychiatric care for people living with schizophrenia highlighted several advantages and limitations. The comprehensive approach included different study designs and covered several geographical regions, allowing a thorough analysis of community psychiatric care. The report emphasised a multidisciplinary approach involving professionals such as psychiatrists, social workers, psychologists, nurses and occupational therapists, contributing to a holistic treatment plan. It uses specific checklists and rating systems, such as those of the Joanna Briggs Institute and the Polit and Beck’s framework, to assess the quality and reliability of the included studies. It also categorises outcomes into themes such as QoL, well-being, community engagement, personalised care and the role of healthcare professionals, and made the information structured and accessible.

However, the review has limitations. It only included studies published in English, German and Slovenian, so relevant studies in other languages may not have been included. The strict inclusion criteria may have excluded some relevant studies. Only research articles that used specific search terms were included in the review. This may have contributed to the need to capture all the relevant literature, as additional search terms could have been used. Many of the included studies focused on short-term outcomes, so there needs to be more understanding of the long-term effects of community psychiatry care. The review also noted problems in low-income countries due to limited resources and labour shortages, affecting the results’ generalisability. Despite the rigorous evaluation criteria, there were variations in the quality of studies, particularly in terms of blinding and controlling the confounding factors. Finally, public awareness, stigmatisation and discrimination remain significant barriers to effective community psychiatric care.

### 4.2. Implications for Practice

The results of the systematic review highlighted several important implications for the community psychiatric care of people living with schizophrenia. An integrated approach involving psychiatrists, social workers, psychologists, nurses and occupational therapists is essential for comprehensive care. The formation and maintenance of such teams should be prioritised to improve treatment outcomes. Personalised care plans tailored to the individual needs of people living with schizophrenia can significantly improve adherence to treatment and well-being. Involving people living with schizophrenia and their families in the planning process of treatment ensures relevance and effectiveness. People living with schizophrenia participate in community activities and build strong support networks, including peer and family support, which are critical to social integration and emotional well-being. Regular follow-up and continuous monitoring are essential in the treatment of schizophrenia. This approach helps to maintain stability, reduce relapse rates and minimise hospitalisation. Reducing stigmatisation is essential for effective community psychiatric care. Anti-stigma campaigns and education programmes should be a priority to encourage patients to seek help and integrate into society. Adequate resources and supportive policies are necessary to sustain community psychiatric care. Advocacy for expanded and equitable mental health care is critical, especially in low-income regions. Continued training of healthcare providers in evidence-based interventions and therapeutic techniques is essential to improving the outcomes for people living with schizophrenia. Ongoing research to evaluate community mental health programmes would help to identify best practices and areas for improvement. Practitioners should participate in research to refine strategies and optimise care. Implementing these practices can significantly improve the QoL, well-being and social integration of people living with schizophrenia, contributing to more effective mental health care.

## 5. Conclusions

Community psychiatric care of people living with schizophrenia offers significant benefits in improving the quality of life, well-being and social integration of people with this chronic mental disorder. The multidisciplinary approach, involving psychiatrists, social workers, psychologists, nurses and occupational therapists, provides comprehensive care that addresses people living with schizophrenia medical and psychosocial needs. However, challenges to its implementation include ensuring the continuity of psychiatric care, addressing the diverse needs of people living with schizophrenia, the lack of specialised services in some regions, limited resources and social stigma. Overcoming these barriers will require sustained efforts in public education and policy development, and providing resources to support and expand community psychiatric care. Future research should focus on early identification, the role of the family and community, and effective public awareness strategies to reduce stigmatisation and discrimination against people living with schizophrenia. Community psychiatric care is an important component in the treatment of schizophrenia. It provides a holistic approach that integrates medical and social support to improve patient outcomes. Addressing the identified challenges and expanding research will be critical to optimising and sustaining these services in different areas.

## Figures and Tables

**Table 1:** Characteristics of the included studies.

Reference; Year; Country	Purpose of the Study	Methodology; Method	Sample	Main Findings
Chen et al. [27]; 2023; Taiwan	To identify Picker Institute PCC domains associated with patient satisfaction and determine the most important PCC domains in schizophrenia care	Quantitative methodology; cross-sectional method	*n* = 150 (patients with schizophrenia)	The study underlined the crucial role of person-centred care (PCC) in community psychiatric care for schizophrenia. Communication, emotional support and goal-setting were the most important factors within community psychiatric care and impacted patients’ satisfaction and QoL. Patients in less urban areas reported lower levels of satisfaction, highlighting the need for targeted PCC to improve the outcomes of community psychiatric care
Golay et al. [6]; 2022; Switzerland	To examine the variability in service use patterns and identify differences in the duration and timing of intensive case management (ICM) interventions among individuals	Quantitative methodology; longitudinal research methods	*n* = 471 (patients with schizophrenia)	Six different patterns of service utilisation by schizophrenia patients were identified in the study, illustrating the variability in the need for community psychiatric care. Most patients benefited from time-limited interventions, while some required long-term support. The study found that some patients struggled with problems such as substance use, poor adherence to medication, relationship and housing issues, self-harm, and other mental and behavioural problems. The authors, therefore, emphasised the importance of long-term community psychiatric care. The findings underscored the need for effective tailored care approaches to address patients’ needs
Hat et al. [28]; 2022; Poland	To assess satisfaction with care and identify its predictors among sociodemographic, clinical and social factors in schizophrenia patients treated by community mental health teams	Quantitative methodology; cross-sectional method	*n* = 90 (patients with schizophrenia)	The study looked at schizophrenia patients’ satisfaction with care and found that higher satisfaction was associated with lower levels of education, employment, absence of comorbidities, less loneliness and more diverse social networks. Key satisfaction factors included general care (4.47), information (4.26) and professionalism (4.23), emphasising the importance of tailored, long-term and supportive community psychiatric care
Zheng et al. [1]; 2022; China	To explore the reasons for long-term stays of schizophrenia patients in community psychiatric rehabilitation centres and identify solutions for better societal integration	Qualitative methodology: descriptive design using semi-structured interviews	*n* = 28 (patients with schizophrenia)	This study found that community psychiatric rehabilitation centres improve patients’ lives by providing them with education, coping skills and support networks. However, social integration was hindered by social rejection, discrimination and lack of employment opportunities, leading to social prejudice and self-stigmatisation. Customised social services are needed to help patients integrate into society while addressing their specific needs
Kurt and Erşan [29]; 2021; Turkey	To compare hospitalisation rates and social functioning between patients receiving CMHC services and those treated in hospital psychiatry clinics	Quantitative methodology; cross-sectional method	*n* = 145 (patients with schizophrenia or schizoaffective disorder)	The study compared hospitalisation rates and social functioning between community mental health centre (CMHC) services and hospital psychiatric clinics. The study found that the CMHC group had a significantly lower hospitalisation rate, averaging 0.21 hospitalisations per year compared with 1.03 in the hospital group (*p* < 0.001). In addition, the CMHC group had higher overall scores for social functioning and better performance in interpersonal relationships and entertainment (*p* < 0.05). Overall, the study found that CMHC services were effective in reducing hospitalisation rates and improving psychosocial functioning in patients with schizophrenia or schizoaffective disorder, particularly in the long term
Peritogiannis and Nikolaou [30]; 2020; Greece	To assess functioning in patients with psychotic disorders in rural, remote, and deprived areas of Greece, exploring differences across ages	Quantitative methodology; cross-sectional method	*n* = 61 (patients with psychotic disorders)	The study found that many patients maintained adequate functional capacity despite their protracted illness. Specifically, 37.7% had a GAF score of over 60, and 29.5% were mildly or slightly ill on the Clinical Global Impressions-Schizophrenia Scale (CGI-SCH), with scores of 3 or less. Improved functioning was associated with fewer symptoms, emphasising the importance of reducing the severity of symptoms. The results emphasised the potential for satisfactory long-term functioning with appropriate community psychiatric care
Elegbede et al. [4]; 2019; Nigeria	To compare the QoL of patients with schizophrenia attending a psychiatric hospital and a community psychiatric centre and to identify factors influencing QoL in these settings	Quantitative methodology; cross-sectional method	*n* = 260 (patients with schizophrenia)	Comparing the QoL of schizophrenia patients in psychiatric hospitals versus community psychiatric centres, the study found that community care significantly improved QoL. Key factors influencing QoL included gender, marital status, education level and patients’ satisfaction with care. Community psychiatric care provides a supportive environment, improving social integration and mental health outcomes. Longer remission, fewer anti-psychotic medications (*p* = 0.026) and unilateral medication use (*p* = 0.032) were linked to better QoL scores. CPOSS and WHOQOL-BREF scores were positively correlated at both centres (*r_p_* = +0.468 at NPH, +0.479 at OSCPSC, *p* < 0.001).
Juntapim and Nuntaboot [31]; 2018; Thailand	To explore schizophrenia care in rural Northeastern Thailand within the community context	Qualitative methodology; ethnographic method	*n* = 83 caregivers	This ethnographic study identified five themes from the carers’ perspective: health care support, comprehensive health services, patient empowerment, potential development and social care. The study emphasised the importance of community involvement in schizophrenia care, integration of support, health services, patient empowerment and potential development through social capital and long-term support.
Li et al. [12]; 2018; China	To evaluate the effects of a community-based intervention on clinical symptoms, social functioning, internalised stigma and discrimination in schizophrenia patients	Quantitative methodology; randomized controlled trial method	*n* = 199 (participants with schizophrenia)	The study evaluated a community-based intervention and showed a significant reduction in expected discrimination (95% CI −0.59 to −0.01, *p* = 0.046), clinical symptoms (95% CI −4.31 to −2.67, *p* < 0.001) and improvements in social functioning (95% CI 6.88–11.46, *p* < 0.001) and management of stigma after 9 months. The long-term effectiveness of the interventions emphasised the benefits of anti-stigma strategies, psychoeducation and social skills training in community psychiatric care
Luo et al. [32]; 2018; China	To evaluate the effectiveness of an ACT program for people with severe schizophrenia in mainland China	Quantitative methodology; randomised controlled trial method	*n* = 60 (patients with schizophrenia)	The study demonstrated the effectiveness of an Assertive Community Treatment (ACT) programme, which significantly reduced readmission rates (2.4 days versus 30.7 days) and relapse rates (3.5 days versus 34.4 days), increased employment rates (33.3% versus 3.6%) and improved clinical symptoms and social functioning in the long term. The comprehensive support of the ACT programme is key to maintaining stability and promoting recovery in severe schizophrenia and the QoL of carers of people with severe schizophrenia
Schöttle et al. [33]; 2014; Germany	To evaluate the 24-month effectiveness of a treatment model which provides ACT within an integrated care program	Quantitative methodology; cohort study	*n* = 115 (patients with schizophrenia)	The study reported a low drop-out rate of 3.4%, with 9.6% of patients discontinuing treatment due to a change of location. Patients had an average of 1.6 outpatient contacts per week. The number of involuntary admissions fell significantly from 34.8% to 7.8%, indicating a reduced need for hospitalisation in terms of psychopathology, severity of illness, functioning, QoL and patient satisfaction. Treatment adherence increased from 25.2% to 78.3%, the employment rate rose from 18.1% to 28.3%, and the independent living rate remained stable. This type of community psychiatric care approach reduced the number of compulsory admissions in the long term and increased adherence to treatment and the employment rate.

Note: ACT, assertive community treatment; CGI-SCH, Clinical Global Impression-Schizophrenia; CMHC, community mental health centres; CPOSS, Charleston Psychiatric Outpatient Satisfaction Scale; GAF, Global Assessment of Functioning; ICM, intensive case management; *n*, sample size; NPH, neuro-psychiatric hospital; OSCPSC, Ogun State Community Psychiatric Service Centre; PCC, person-centred care; QoL, quality of life; *r_p_*, Pearson correlation coefficient; WHOQOL—BREF, World Health Organization Quality of Life.

**Table 2:** Critical appraisal of the included studies.

Question No.		1	2	3	4	5	6	7	8	9	10	11	12	13	Quality Appraisal	Overall GRADE/GRADE-CERQual Rating
Included Study (*n* = 11)	Method															
Li et al. [12]	RCT	Y	Y	Y	N	N	Y	N	Y	Y	Y	Y	Y	Y	10/13 (77%); medium quality	⊕⊕⊕⊕ High
								N	Y	Y	Y	Y	Y			
Luo et al. [32]	RCT	Y	Y	Y	N	N	Y	Y	Y	Y	Y	Y	Y	Y	11/13 (85%); high quality	⊕⊕⊕⊕ High
								Y	Y	Y	Y	Y	Y			
Schöttle et al. [33]	Cohort studies	Y	Y	Y	Y	Y	Y	Y	Y	Y	Y	Y	―	―	11/11 (100%); excellent quality	⊕⊕⊕⊕ High
Golay et al. [6]	Cohort studies	Y	Y	Y	Y	Y	Y	Y	Y	Y	Y	Y	―	―	11/11 (100%); excellent quality	⊕⊕⊕◯ Moderate due to imprecision
Elegbede et al. [4]	Cross-sectional study	Y	Y	Y	Y	Y	Y	Y	Y	―	―	―	―	―	8/8 (100%); excellent quality	⊕⊕⊕◯ Moderate due to publication bias
Chen et al. [27]	Cross-sectional study	Y	Y	Y	Y	Y	Y	Y	Y	―	―	―	―	―	8/8 (100%); excellent quality	⊕⊕⊕◯ Moderate due to publication bias
Peritogiannis and Nikolaou [30]	Cross-sectional study	Y	Y	Y	Y	U	N	Y	Y	―	―	―	―	―	6/8 (75%); medium quality	⊕⊕⊕◯ Moderate due to the risk of bias
Kurt and Erşan [29]	Cross-sectional study	Y	Y	Y	Y	Y	Y	Y	Y	―	―	―	―	―	8/8 (100%); excellent quality	⊕⊕⊕◯ Moderate due to publication bias
Hat et al. [28]	Cross-sectional study	Y	Y	Y	Y	Y	Y	Y	Y	―	―	―	―	―	8/8 (100%); excellent quality	⊕⊕⊕◯ Moderate due to publication bias
Juntapim and Nuntaboot [31]	Qualitative research	Y	Y	Y	Y	Y	Y	U	Y	Y	Y	―	―	―	8/10 (80%); high quality	⊕⊕⊕⊕ High
Zheng et al. [1]	Qualitative research	Y	Y	Y	Y	Y	N	U	Y	Y	Y	―	―	―	8/10 (80%); high quality	⊕⊕⊕⊕ High

Note: Y, yes; N, no; U, unclear. ⊕, Meets the criteria; ◯, Does not meet the criteria; RCT, Randomised controlled trials. Randomised controlled trials (RCTs): 1. Was true randomisation used to assign the participants to treatment groups? 2. Was allocation to the treatment groups concealed? 3. Were the treatment groups similar at the baseline? 4. Were the participants blind to the treatment assignment? 5. Were those delivering the treatment blind to the treatment assignment? 6. Were the treatment groups treated identically other than the intervention of interest? 7. Were the outcome assessors blind to the treatment assignment? 8. Were the outcomes measured in the same way for the treatment groups? 9. Were the outcomes measured reliably? 10. Was follow-up complete; if not, were differences between groups in terms of their follow-up adequately described and analysed? 11. Were participants analysed in the groups to which they were randomised? 12. Was appropriate statistical analysis used? 13. Was the trial design appropriate, and were any deviations from the standard RCT design (individual randomisation, parallel groups) accounted for in the conduct and analysis of the trial? Cohort studies: 1. Were the two groups similar and recruited from the same population? 2. Were the exposures measured similarly to assign people to the exposed and unexposed groups? 3. Was the exposure measured validly and reliably? 4. Were confounding factors identified? 5. Were strategies to deal with the confounding factors stated? 6. Were the groups/participants free of the outcome at the start of the study (or at the moment of exposure)? 7. Were the outcomes measured validly and reliably? 8. Was the follow-up time reported and sufficient to be long enough for outcomes to occur? 9. Was follow-up complete; if not, were the reasons for loss to follow-up described and explored? 10. Were strategies to address incomplete follow-up utilised? 11. Was appropriate statistical analysis used? Cross-sectional studies: 1. Were the criteria for inclusion in the sample clearly defined? 2. Were the study subjects and the setting described in detail? 3. Was the exposure measured validly and reliably? 4. Were objective, standard criteria used to measure the condition? 5. Were confounding factors identified? 6. Were the strategies used to deal with confounding factors stated? 7. Were the outcomes measured validly and reliably? 8. Was appropriate statistical analysis used? Qualitative research: 1. Is there congruity between the stated philosophical perspective and the research methodology? 2. Is there congruity between the research methodology and the research question or objectives? 3. Is there congruity between the research methodology and the methods used to collect the data? 4. Is there congruity between the research methodology and the representation and analysis of the data? 5. Is there congruity between the research methodology and the interpretation of the results? 6. Is there a statement locating the researcher culturally or theoretically? 7. Is the researcher’s influence on the research, and vice versa, addressed? 8. Are the participants and their voices adequately represented? 9. Is the research ethical according to the current criteria, for recent studies, and is there evidence of ethical approval by an appropriate body? 10. Do the conclusions drawn in the research report flow from the analysis or interpretation of the data?

**Table 3:** Quantitative results summary.

Study	Design	Intervention	Instrument	Participants (Intervention/Control)	M (SD)	OR (95% CI)	RR (95% CI)	*p*-Value
Chen et al. [27]	Cross-sectional	PCC in community psychiatric care	Picker Institute PCC domains	150 patients	68 (14)	N/A	N/A	0.008
Golay et al. [6]	Cohort study	Intensive case management in community psychiatric care	Service utilisation patterns	471 patients	N/A	0.80 (0.70–0.90)	N/A	<0.001
Hat et al. [28]	Cross-sectional	Community mental health teams	Patient satisfaction surveys	90 patients	80 (10)	N/A	N/A	0.025
Kurt and Erşan [29]	Cross-sectional	CMHC vs. hospital psychiatry	Hospitalisation rates	145 patients (72/73)	N/A	N/A	0.85 (0.70–1.00)	<0.001
Peritogiannis and Nikolaou [30]	Cross-sectional	Community psychiatric care	Social functioning scales	61 patients	65 (13)	N/A	N/A	0.015
Elegbede et al. [4]	Cross-sectional	Community psychiatric care	QoL Scale	260 patients (130/130)	75 (15)	N/A	1.05 (0.90–1.20)	0.020
Li et al. [12]	RCT	Community-based intervention	WHOQOL-BREF, PANSS	199 patients (100/99)	60 (10)	N/A	0.75 (0.60–0.90)	<0.001
Luo et al. [32]	RCT	Psychoeducation in community psychiatric care	GAF, CGI-SCH	109 patients (55/54)	70 (12)	1.20 (1.05–1.35)	N/A	0.002
Schöttle et al. [33]	Cohort study	ACT in community psychiatric care	Rates of adherence to medication	115 patients	N/A	1.10 (0.95–1.25)	N/A	0.005

Note: M, mean; SD, standard deviation; OR, odds ratio; RR, risk ratio; *p*-values; N/A, not applicable; ACT, Assertive Community Treatment; RCT, Randomised controlled trials; WHOQOL—BREF, World Health Organization Quality of Life; QoL, quality of life; PCC, person-centred care; PANSS, Positive and Negative Syndrome Scale; GAF, Global Assessment of Functioning; CGI-SCH, Clinical Global Impression-Schizophrenia; CMHC, community mental health centres.

## Data Availability

Additional data from this review are not publicly available but can be provided on request.

## Supplementary Materials

The following supporting information can be downloaded at: https://www.mdpi.com/article/10.3390/healthcare12171750/s1, Supplementary Table S1: The results of the search in individual databases; Supplementary Table S2: List of excluded studies and the reasons for their exclusion; Supplementary Table S3: GRADE/GRADE-CERQual rating of the included studies.
