# Supplementary material for: Long-Term Impact of Community Psychiatric Care on Quality of Life amongst People Living with Schizophrenia: A Systematic Review
Source: Healthcare (Basel). 2024 Sep 2;12(17):1750. doi: 10.3390/healthcare12171750 (PMC11394810; doi:10.3390/healthcare12171750)
Supplement: Supplementary file 1 [file healthcare-12-01750-s001.zip › healthcare-3118766-supplementary.pdf]

Supplementary Table S1: The results of the search in individual databases

	CINAHL	
#	Searches	Records
1	(meaning* OR importance* OR impact*)	673,965
2	("community psychiatry*" OR "community psychiatric")	7,124
3	(treatment OR care OR service)	2,489,506
4	(patient OR (people OR individual))	2,628,185
5	carer	35,385
6	schizophrenia	38,078
7	#1 AND #2	998
8	#1 AND #2 AND #3	985
9	#1 AND #2 AND #3 AND #4	666
10	#1 AND #2 AND #3 AND #4 AND #5	35
11	#1 AND #2 AND #3 AND #4 AND #5 AND #6	3
Search limit use		
Time-frame:	Until March 2024	
Language:	English, German and/or Slovenia	
Types of article:	Research paper	

	Medline	
#	Searches	Records
1	(meaning* OR importance* OR impact*)	2,671,152
2	("community psychiatry*" OR "community psychiatric")	19,649
3	(treatment OR care OR service)	8,972,210
4	(patient OR (people OR individual))	9,744,684
5	carer	45,243
6	schizophrenia	31,017
7	#1 AND #2	1,985
8	#1 AND #2 AND #3	1,839
9	#1 AND #2 AND #3 AND #4	1,243
10	#1 AND #2 AND #3 AND #4 AND #5	61
11	#1 AND #2 AND #3 AND #4 AND #5 AND #6	9
Search limit use		
Time-frame:	Until March 2024	
Language:	English, German and/or Slovenia	
Types of article:	Research paper	

	Sage	
#	Searches	Records
1	(meaning* OR importance* OR impact*)	1,171,880
2	("community psychiatry*" OR "community psychiatric")	2,512
3	(treatment OR care OR service)	1,589,123

4	(patient OR (people OR individual))	1,592,892
5	carer	627,867
6	schizophrenia	32,061
7	#1 AND #2	1,884
8	#1 AND #2 AND #3	32,061
9	#1 AND #2 AND #3 AND #4	1,875
10	#1 AND #2 AND #3 AND #4 AND #5	1,651
11	#1 AND #2 AND #3 AND #4 AND #5 AND #6	756
Search limit use		
Time-frame:	Until March 2024	
Language:	English, German and/or Slovenia	
Types of article:	Research paper	

	ScienceDirect	
#	Searches	Records
1	(meaning OR importance)	1,000,000+
2	("community psychiatry"OR "community psychiatric")	3,248
3	(treatment OR care)	1,000,000+
4	patient	1,000,000+
5	carer	1,000,000+
6	schizophrenia	105,639
7	#1 AND #2	1,862
8	#1 AND #2 AND #3	1,644
9	#1 AND #2 AND #3 AND #4	1,529
10	#1 AND #2 AND #3 AND #4 AND #5	1,529
11	#1 AND #2 AND #3 AND #4 AND #5 AND #6	753
Search limit use		
Time-frame:	Until March 2024	
Language:	English, German and/or Slovenia	
Types of article:	Research paper	

	Web of Science	
#	Searches	Records
1	(meaning* OR importance* OR impact*)	4,996,679
2	("community psychiatry*" OR "community psychiatric")	4,159
3	(treatment OR care OR service)	8,604,769
4	(patient OR (people OR individual))	8,962,641
5	carer	18,821
6	schizophrenia	140,383
7	#1 AND #2	66
8	#1 AND #2 AND #3	49
9	#1 AND #2 AND #3 AND #4	162
10	#1 AND #2 AND #3 AND #4 AND #5	7
11	#1 AND #2 AND #3 AND #4 AND #5 AND #6	4
Search limit use		
Time-frame:	Until March 2024	
Language:	English, German and/or Slovenia	

Supplementary Table S2: List of excluded studies and the reasons for their exclusion

No.	Reference	Reason
1.	Asher, Laura et al. "Community-based rehabilitation intervention for people with schizophrenia in Ethiopia (RISE): results of a 12-month cluster-randomized controlled trial." The Lancet. Global health vol. 10,4 (2022): e530-e542.	Inadequate population.
2.	Zhu, Xiaolin et al. "Improving social functioning in community-dwelling patients with schizophrenia: a randomised controlled computer cognitive remediation therapy trial with six months follow-up." Psychiatry Research vol. 287 (2020): 112913.	Does not relate to the review's focus or research question.
3.	Gowda, Guru S, and Mohan K Isaac. "Models of Care of Schizophrenia in the Community-An International Perspective." Current Psychiatry Reports vol. 24,3 (2022): 195-202.	Does not relate to the review's focus or research question.
4.	Munikanan, Thurkadevi et al. "Association of social support and quality of life among people with schizophrenia receiving community psychiatric service: A cross-sectional study." Comprehensive Psychiatry vol. 75 (2017): 94-102.	Does not relate to the review's focus or research question.
5.	Park, Suin et al. "Nurse Staffing and Health Outcomes of Psychiatric Inpatients: A Secondary Analysis of National Health Insurance Claims Data." Journal of Korean Academy of Nursing vol. 50,3 (2020): 333-348.	Does not relate to the review's focus or research question.
6.	Ryan, Denis, and Jane Alexander. "Mental health nursing in community care." Psychiatric and Mental Health Nursing. Routledge, 2017. 607-618.	Inadequate type of article.
7.	Parry, S.L., Eve, Z., Stamou, V., Brockway, A. and Di Basilio, D. (2023), "“Friendly, local and welcoming” – evaluation of a community mental health early intervention service", Journal of Children's Services, Vol. 18 No. 3/4, pp. 233-243.	Does not relate to the review's focus or research question.
8.	Puntis, Stephen et al. "Specialised early intervention teams for recent-onset psychosis." The Cochrane Database of Systematic Reviews vol. 11,11 CD013288. 2 Nov. 2020.	Inadequate type of article.
9.	Burns, Tom et al. "Effect of increased compulsion on readmission to hospital or disengagement from community services for patients with psychosis: follow-up of a cohort from the OCTET trial." The Lancet. Psychiatry vol. 2,10 (2015): 881-90.	Does not relate to the review's focus or research question.
10.	Zhang, Wufang et al. "Effect of a contact-based education intervention on reducing stigma among community health and care staff in Beijing, China: Pilot randomised controlled study." Asian Journal of Psychiatry vol. 73 (2022): 103096.	Does not relate to the review's focus or research question.
11.	Ran, Mao-Sheng et al. "Family history of psychosis and outcome of people with schizophrenia in rural China: 14-year follow-up study." Asian Journal of Psychiatry vol. 32 (2018): 14-19.	Does not relate to the review's focus or research question.
12.	Arraras, J. I., Ibañez, B., Basterra, I., Pereda, N., Martin, M., & Iribarren, S. (2018). Determinants of quality of life in Spanish outpatients with schizophrenia spectrum disorders. The European Journal of Psychiatry, 32(3), 113–121.	Does not relate to the review's focus or research question.

No.	Reference	Reason
13.	Stanga, Valentina et al. "Well-being in patients with schizophrenia, mood and personality disorders attending psychiatric services in the community. A controlled study." Comprehensive Psychiatry vol. 91 (2019): 1-5.	Does not relate to the review's focus or research question.
14.	Kane, John M et al. "Comprehensive Versus Usual Community Care for First-Episode Psychosis: 2-Year Outcomes From the NIMH RAISE Early Treatment Program." The American Journal of Psychiatry vol. 173,4 (2016): 362-72.	Does not relate to the review's focus or research question.
15.	Malone, D. et al. "Community mental health teams (CMHTs) for people with severe mental illnesses and disordered personality." The Cochrane Database of Systematic Reviews vol. 2007,3 CD000270.	Inadequate type of article.
16.	Chatterjee S, Naik S, John S, Dabholkar H, Balaji M, Koschorke M, Varghese M, Thara R, Weiss HA, Williams P, McCrone P, Patel V, Thornicroft G. Effectiveness of a community-based intervention for people with schizophrenia and their caregivers in India (COPSI): a randomised controlled trial. Lancet. 2014 Apr 19;383(9926):1385-94.	Does not relate to the review's focus or research question.
17.	Ertekin Pinar, Sukran, and Selma Sabanciogullari. "The relationship between functional recovery and quality of life in patients affected by schizophrenia and treated at a community mental health centre in Turkey." Perspectives in psychiatric care vol. 56,2 (2020): 448-454.	Does not relate to the review's focus or research question.

Supplementary Table S3: GRADE / GRADE-CERQual rating of the included studies

GRADE criteria		Risk of Bias	Inconsistency	Indirectness	Imprecision	Publication Bias	Overall GRADE / GRADE-CERQual Rating
**Included paper (*n* = 9)**	**Method**						
Li, Huang [12]	RCT	⊕	⊕	⊕	⊕	⊕	⊕⊕⊕⊕<br>High
Luo, Law [32]	RCT	⊕	⊕	⊕	⊕	⊕	⊕⊕⊕⊕<br>High
Schöttle, Schimmelmann [33]	Cohort studies	⊕	⊕	⊕	⊕	⊕	⊕⊕⊕⊕<br>High
Golay, Bonsack [6]	Cohort studies	⊕	⊕	⊕	⊖	⊕	⊕⊕⊕○<br>Moderate due to the imprecision
Elegbede, Obadeji [4]	Cross-sectional study	⊕	⊕	⊕	⊕	⊖	⊕⊕⊕○<br>Moderate due to the publication bias
Chen, Chueh [27]	Cross-sectional study	⊕	⊕	⊕	⊕	⊖	⊕⊕⊕⊖<br>Moderate due to the publication bias
Peritogiannis and Nikolaou [30]	Cross-sectional study	⊖	⊕	⊕	⊕	⊕	⊕⊕⊕○<br>Moderate due to the risk of bias
Kurt and Erşan [29]	Cross-sectional study	⊕	⊕	⊕	⊕	⊖	⊕⊕⊕⊖<br>Moderate due to the publication bias
Hat, Arciszewska-Leszczuk [28]	Cross-sectional study	⊕	⊕	⊕	⊕	⊖	⊕⊕⊕○

GRADE criteria		Risk of Bias	Inconsistency	Indirectness	Imprecision	Publication Bias	Overall GRADE / GRADE-CERQual Rating
**Included paper (*n* = 9)**	**Method**						
							Moderate due to the publication bias

GRADE-CERQual criteria		Methodological limitations	Coherence	Adequacy of component	Relevance	Overall GRADE / GRADE-CERQual Rating
**Included paper (*n* = 2)**	**Method**					
Juntapim and Nuntaboot [31]	Qualitative research	⊕	⊕	⊕	⊕	⊕⊕⊕⊕ High
Zheng, Zhang [1]	Qualitative research	⊕	⊕	⊕	⊕	⊕⊕⊕⊕ High
